# GVHD Prophylaxis 2020

**DOI:** 10.3389/fimmu.2021.605726

**Published:** 2021-04-07

**Authors:** Mahasweta Gooptu, Joseph Harry Antin

**Affiliations:** Dana–Farber Cancer Institute, Boston, MA, United States

**Keywords:** GvHD prophylaxis, calcineurin inhibitors, post-transplant cyclophosphamide, T-cell depletion, T-cell modulation, T-cell trafficking

## Abstract

Graft-vs. host disease (GVHD), both acute and chronic are among the chief non-relapse complications of allogeneic transplantation which still cause substantial morbidity and mortality despite significant advances in supportive care over the last few decades. The prevention of GVHD therefore remains critical to the success of allogeneic transplantation. In this review we briefly discuss the pathophysiology and immunobiology of GVHD and the current standards in the field which remain centered around calcineurin inhibitors. We then discuss important translational advances in GVHD prophylaxis, approaching these various platforms from a mechanistic standpoint based on the pathophysiology of GVHD including *in-vivo* and *ex-vivo* T-cell depletion alongwith methods of selective T-cell depletion, modulation of T-cell co-stimulatory pathways (checkpoints), enhancing regulatory T-cells (Tregs), targeting T-cell trafficking as well as cytokine pathways. Finally we highlight exciting novel pre-clinical research that has the potential to translate to the clinic successfully. We approach these methods from a pathophysiology based perspective as well and touch upon strategies targeting the interaction between tissue damage induced antigens and T-cells, regimen related endothelial toxicity, T-cell co-stimulatory pathways and other T-cell modulatory approaches, T-cell trafficking, and cytokine pathways. We end this review with a critical discussion of existing data and novel therapies that may be transformative in the field in the near future as a comprehensive picture of GVHD prophylaxis in 2020. While calcineurin inhibitors remain the standard, post-transplant eparinsphamide originally developed to facilitate haploidentical transplantation is becoming an attractive alternative to traditional calcinuerin inhibitor based prophylaxis due to its ability to reduce severe forms of acute and chronic GVHD without compromising other outcomes, even in the HLA-matched setting. In addition T-cell modulation, particularly targeting some important T-cell co-stimulatory pathways have resulted in promising outcomes and may be a part of GVHD prophylaxis in the future. Novel approaches including targeting early events in GVHD pathogenesis such as interactions bvetween tissue damage associated antigens and T-cells, endothelial toxicity, and T-cell trafficking are also promising and discussed in this review. GVHD prophylaxis in 2020 continues to evolve with novel exicitng therapies on the horizon based on a more sophisticated understanding of the immunobiology of GVHD.

## Introduction

GVHD prophylaxis has come a long way since the initial days of allogeneic transplantation. The improvement in GVHD outcomes has been one of the primary reasons for the reduction in non-relapse mortality over time (1) that has enhanced the success of allogeneic transplantation and allowed us to perform transplants in older patients as well as those with co-morbidities. GVHD comprises two distinct entities-acute GVHD (aGVHD) which typically presents in the first 3–6 months following transplant and manifests as a characteristic rash, secretory diarrhea, or cholestatic liver function abnormalities and chronic GVHD (cGVHD) which presents usually after the first 3 months and can affect virtually any organ system (ocular, oral, skin, musculo-skeletal, gastro-intestinal, pulmonary etc.,). Overlap syndromes are well-recognized, although relatively rare. These two entities have distinct pathophysiologies as well, however, prophylactic strategies generally try to prevent both acute and chronic varieties albeit with varying success depending on the strategy.

In HLA-matched transplantation, while the backbone of most widely used prophylactic platforms remains calcineurin-inhibitor (CNI)-based, a variety of new drugs have been added to CNI's in an attempt to improve efficacy and reduce toxicity. In this review, we discuss current standards and their evolution over time and highlight some of these translational advances. Further we touch upon novel pre-clinical advances developed on the foundation of a deeper understanding of transplant immunology and promising for translation to the clinic. We begin with a description of the immunobiology of GVHD, to better understand potential targets which have been exploited over the last few decades and currently to develop effective prophylactic therapies for GVHD.

### The Immunobiology of Graft-vs.-Host Disease

#### Acute GVHD

One of the first models describing the biology of GVHD was proposed by Antin and Ferrara where they described a sequential cascade initiated by conditioning regimen mediated host tissue injury with the production of inflammatory cytokines (phase 1). This is followed by activation and proliferation of effector T-lymphocytes (phase II) which eventually lead to recruitment and activation of additional mononuclear effectors and amplification of a “cytokine storm” (Phase III) (2). Further extensive research has refined these concepts and identified targets for development of novel prophylactic strategies to prevent acute and chronic GVHD.

##### Phase 1

Both neutrophils and monocytes are involved in the initial inflammatory response in the pathogenesis of GVHD. Monocytes are activated by molecules called damage-associated molecular patterns (DAMPs) such as uric acid, ATP, Heparan sulfate, HMGB-1 or IL-33 which can initiate and perpetuate a non-infectious inflammatory response involving the innate immune system. In contrast pathogen-associated molecular patterns (PAMPs) such as bacterial lipopolysaccharides cause infection-associated inflammation. The DAMP and PAMP mediated inflammatory responses result in activation of the innate immune system (monocytes and neutrophils) which then cause local tissue damage mediated by reactive oxygen species. This eventually culminates in interaction of antigen-presenting cells (APCs) in the innate and adaptive immune and activation of cytokine cascades (IL-1, IL-6, TNF-α, etc.,) leading to the “cytokine storm (3).” Prophylactic strategies targeting these events have focused on arresting the cytokine storm through inhibition of particular cytokines or interrupting the interaction between APCs and PAMP.

##### Phase II

Primed by this cytokine storm, effector T-lymphocytes now migrate to lymphoid organs and host tissues mediated by L-selectin, CCR7. This culminates in APC mediated T-cell activation and engagement of the T-cell receptor complex and modulation by anti and co-stimulatory pathways. In particular, T-lymphocytes trafficking to the gut express high levels of integrin β7 (α4β7) which bind corresponding host tissue ligands presenting a potential target for intervention. The T-cell activation process and proliferation process is a crucial target for GVHD prevention.

##### Phase III

These events lead to a self-potentiating T-lymphocyte activation causing tissue damage via direct cellular cytotoxicity and indirectly via release of soluble mediators (TNF-α, IFN-γ, IL-1, and nitric oxide) (4).

A number of additional pathways have since been implicated in GVHD pathogenesis including the canonical NOTCH pathway (5). It has been shown via monoclonal antibodies that Notch-deprived T-cells proliferate normally but produce less inflammatory cytokines with a preferential increase in Tregs (6) and all the effects were dependent on NOTCH1/2 receptors on T-cells and Dll1/4 ligands in the recipient with dominant roles for NOTCH1 and Dll4 (6).

While the pro-inflammatory signals described above potentiate GVHD, there are also anti-inflammatory components of the immune system that try to dampen these inflammatory responses. Regulatory T-cells (Tregs) are important in immunologic tolerance, partly *via* release of anti-inflammatory cytokines such as IL-10 and TGF-β (7). Cytokine responses are often classified as effector T helper (Th) type 1 (IL-2, INF-γ) and type 2 (IL-4, IL-10) responses where type 2 cytokines can inhibit potent proinflammatory type 1cytokines, and a Th1 to Th2 shift could be beneficial in aGVHD (8). In addition a particular subset of CD4+ cells called Th17 cells have been identified which are characterized by the production characterized by production of *IL-17A* and F, IL-21, and IL-22 and which in murine models migrate to GVHD target organs causing severe pulmonary and GI lesions and GVHD deaths (9). These are postulated to be anatagonistic to Tregs (10) making them an interesting target. Invariant natural killer T (iNKT) cells are another cellular subset with putative immunoregulatory functions, in part via an increase Treg numbers and IL-4 secretion, that may be important in GVHD pathophysiology.

#### Chronic GVHD

Chronic GVHD remains the most common late toxicity of allogeneic transplantation with significant morbidity and quality of life implications. cGVHD has its own distinctive immunobiology. Briefly we can conceptualize the pathophysiology of cGVHD in three phases: (1) Inflammation leading to tissue damage (2) chronic inflammation, thymic injury, dysregulated B- and T-cell immunity (3) tissue repair with fibrosis (11, 12). Although a more detailed discussion of these phases is beyond the scope of this review, we will focus on some of the known interventions that can prevent or reduce the incidence of cGVHD as well as some novel therapies being tested, particularly those targeting the B-cell axis.

Potential targets for developing novel prophylactic platforms have been identified based on our current and more comprehensive understanding of the biology of GVHD. In this review we discuss both current standards and important translational advances as well as exciting new potential therapies which may be translated to the clinic in the future.

### Current Standards in GVHD Prophylaxis

The effective prevention of GVHD is critical to the success of allogeneic transplantation. Based on the understanding that aGVHD is primarily mediated by effector T-lymphocytes, prophylactic strategies have focused on T-cell suppression in the recipient. Calcineurin inhibitors (tacrolimus/Tac and cyclosporine/CyA) inhibit the proliferation and activation of T-cells and have been used in combination with either methotrexate (MTX) or mycophenolate mofetil (MMF) as standard prophylaxis in HLA-matched HSCT. In two randomized controlled trials (RCT) in the 1990s, the combination of Tac/MTX was found to be significantly superior to CyA/MTX is the prevention of grade II-IV aGVHD and extensive chronic GVHD in HLA-matched sibling and unrelated donors, although a benefit in overall survival (OS) was not shown (13, 14). Furthermore, a single-center phase II RCT compared Tac/MTX with Tac/MMF and found that Tac/MTX was more effective in preventing severe aGVHD, particularly in matched unrelated donor (MUD) transplantation (15). CNI based prophylaxis remains the standard in HLA-matched transplantation. However, the recent advent of post-transplant cyclophosphamide (PTCy), has been revolutionary, not only allowing related donor haploidentical transplants to be performed but also making some inroads in the field of HLA-matched transplantation.

### Translational Advances in GVHD Prophylaxis

#### *In-vivo* T-Cell Depletion/Modulation

T-cell depletion or modulation *in-vivo* has been the basis for the development of a number of novel GVHD prophylaxis strategies. These have typically been incorporated into regimens where the backbone comprises CNIs. We summarize some of these approaches below.

##### Post-transplant Cyclophosphamide

Transplantation across HLA barriers historically has been difficult due to high rates of graft rejection and severe GVHD secondary to strong bidirectional alloreactive responses between donor and recipient. The introduction of post-transplant cyclophosphamide (PTCy) in the context of haploidentical transplantation has been a gamechanger and allowed us to perform such mismatched transplants safely and effectively, typically from related donors.

First pioneered at Johns Hopkins, cyclophosphamide is given at doses of 50 mg/kg on days +3 and +4 following the infusion of haploidentical stem-cells. In initial studies reported by the Hopkins group, reduced intensity HSCT with PTCy along with Tac and MMF as GVHD prophylaxis resulted in engraftment in 87% of patients with acceptable rates of grade II-IV (34%) and III-IV aGVHD (6%). Further, rates of chronic severe GVHD were found to be particularly low. Relapse rates were in the 50% range (16). Numerous subsequent studies have shown similar numbers and more recently myeloablative haploidentical transplantation with PTCy based prophylaxis has also been widely adopted (17–19). It is interesting to note that the rates of grade II-IV aGVHD are not in fact significantly lower with PTCy based prophylaxis in haplotransplants compared with those seen in HLA-matched transplant with CNI based prophylaxis and the actual mechanistic implications of PTCy are being investigated.

The earlier more simplistic hypotheses postulated that PTCy results in the deletion of alloreactive T-cells with some debate regarding the effect it may have on the T-cell mediated graft-vs. leukemia (GVL) effect. However, more recently it has been shown in mice that Tregs are preserved and that T-effector cell exhaustion may play an important role (20). A comprehensive model has however not been defined yet.

The beneficial effect of PTCy on severe aGVHD and cGVHD has led to its adoption into the HLA-matched setting more recently. In a Phase II RCT (BMT CTN 1203) which compared three different GVHD prophylaxis regimens in RIC MUD HSCT (PTCy/Tac/MMF, Tac/MTX/bortezomib, and Tac/MTX/maraviroc) with standard Tac/MTX prophylaxis, the PTCy arm fared the best with comparable grade II-IV aGVHD (27%) but lower rates of grade III-IV aGVHD (2%) and cGVHD requiring immunosuppression (22%) outcomes. Relapse rates in the PTCy arm were 28%. Overall GVHD and relapse free survival (GRFS) was superior in the PTCy arm meeting the primary end-point of the trial (21). Again in an RCT from Europe, PTCy/Tac/MMF based prophylaxis fared better than CyA/MMF in the prevention of acute and chronic GVHD in HLA_matched RIC HSCT although the standard of care arm did not include Tac or MTX and numbers were limited (22). Some centers have been using low-dose ATG along with low-dose PTCy to try and improve grade II-IV aGVHD rates (23), however, this is not an universally accepted practice at this time due to limited data.

Bolstered by this data, PTCy/Tac/MMF is now being compared to standard Tac/MTX prophylaxis in RIC HLA-matched HSCT in a large phase III RCT (BMT CTN 1703). PTCy does have the potential to eliminate the effect of donor mismatch on GVHD outcomes, however, CNI based prophylaxis remains the standard until phase III data is available.

##### Anti-thymocyte Globulin

The polyclonal immunoglobulin product obtained from the sera of rabbits and horses immunized with human thymocytes or T-cell lines is called anti-thymocyte globulin or ATG. ATG has been used as part of transplant conditioning to effect *in-vivo* TCD with the aim to reduce both acute and chronic GVHD with varying success.

Of the four RCTs that have evaluated ATG in combination with standard CNI/MTX prophylaxis, the first used horse ATG and showed a reduction in aGVHD; however, there were higher rates of infection with resultant no difference in NRM or OS. Importantly, there was a reduction in chronic severe GVHD (24, 25). The second RCT used rabbit ATG while the third mainly used PBSC grafts. In both of these trials, although there was no effect on aGVHD, a reduction in chronic GVHD was seen once again (26). Therefore, it seems that the use of ATG can reduce severe chronic GVHD with no deleterious effect on OS, however, aGVHD is not consistently reduced.

Rabbit ATG is generally considered to deplete T-cells more effectively as well as allow greater expansion of regulatory T-cells (Tregs) (27). The beneficial effect of ATG on extensive chronic GVHD was suggested in an retrospective analysis which compared ATG to no ATG containing GVHD prophylaxis regimens in matched unrelated donor transplantation (28). Subsequently, in a recent RCT which evaluated Tac/MTX ± anti T-lymphocyte globulin (ATLG), a form of rabbit ATG, in myeloablative unrelated donor transplantation, a significant reduction in grade II-IV aGVHD and moderate/severe chronic GVHD was seen. However, NRM and OS was impaired in the ATLG arm (29). It was suggested that a higher dose of ATLG in the trial may have contributed to increased infections and mortality.

In this context, there is evidence that increased doses or prolonged dosage schedules of ATG may have immunosuppressive toxicity with increased NRM and relapse (30). Individualized ATG dosing, based not just on weight but on absolute lymphocyte count has been proposed as a way to tailor doses of ATG for maximal benefit (31). Different doses of ATG have also been explored in the context of haploidentical transplantation (32).

In the realm of matched related donor transplantation, a recent multi-center randomized study from China demonstrated improved acute and chronic GVHD rates without compromising relapse or treatment-related mortality (33) and merits further study.

##### Sirolimus

Sirolimus is a mTOR inhibitor which inhibits effector T-lymphocytes and in *in-vitro* studies appeared to spare regulatory T-lymphocytes. A favorable ratio of Tregs:Teff has been shown to be associated with better GVHD outcomes and hence sirolimus has an immunologic profile that was thought to be potentially beneficial for GVHD prevention. In addition, it has a distinct toxicity profile compared to tacrolimus and is not nephrotoxic. In a large RCT in myeloablative transplants with HLA-matched donors, sirolimus in combination with tacrolimus was compared with the standard Tac/MTX platform. There was no difference in grades II-IV aGVHD and cGVHD, but better grade III-IV aGVHD outcomes with sirolimus/Tac were seen. Non-relapse mortality (NRM) and OS were similar as well (34). Hence sirolimus appears to be an acceptable alternative to MTX when used with CNIs. In subsequent studies, sirolimus has been associated with higher rates of veno-occlusive disease (VOD) particularly when ablative busulfan is used (35) or when there are additional risk factors for VOD. In RIC transplantation, the addition of sirolimus to Tac/MTX resulted in better grade II-IV aGVHD outcomes without survival benefit in a phase II RCT (36). More recently, a phase II RCT found that the combination of sirolimus with CyA and MMF was superior to CyA/MMF; however, the comparator arm is generally considered inferior to Tac/MTX (37).

Sirolimus has been found to be particularly helpful in situations where nephrotoxicity is a concern such as in transplantation for sickle-cell disease. It is also being used with PTCy in patients with borderline renal function, with rates of engraftment and GVHD comparable to PTCy based regimens with CNI and may be a way to safely perform HSCT in patients with renal dysfunction (38).

Given its Treg sparing effects, novel combinations such as that with OX40L blockade are being investigated (39). This is discussed in greater detail in the section on OX40L blockade later in this review.

#### *Ex-vivo* T-Cell Depletion/Modulation

*Ex-vivo* TCD has been used for decades in allogeneic transplantation as a prophylactic strategy to prevent GVHD. Methods of T-cell depletion have included (1) negative selection (removal of T-lymphocytes) through the use of monoclonal antibodies with or without complement (40, 41), counter flow elutriation (42), and immunotoxins (43) or (2) positive selection of CD34+ hematopoietic stem cells from the graft which (currently the preferred method) usually via immunomagnetic beads with the CliniMACS CD34 Reagent System (Miltenyi Biotech, Gladbach, Germany) (44). The two methods do differ in efficacy with greater TCD being achieved by positive selection.

##### Pan T-Cell Depletion

An early concern with TCD was that it could affect the powerful GVL effect in HSCT which is also believed to be T-cell mediated. In a RCT in patients transplanted with marrow grafts, TCD was compared to conventional prophylaxis with CyA/MTX; the 3-year disease free survival (DFS) was similar in both groups with lower rates of grade III-IV aGVHD with TCD. Relapse rates were however, higher with TCD, particularly in patients with chronic myelogenous leukemia (CML) (45). A large registry analysis also showed higher relapse rates with TCD (46). Subsequently, in a phase II trial with peripheral blood stem-cell (PBSC) transplantation (BMT CTN 0303), immunomagnetic beads were used for CD34 selection of the graft and TCD and relapse rates appeared to be comparable to historic controls while rates of acute and extensive chronic GVHD were favorable (47). Another trial comparing CD34 selected HLA-matched sibling HSCT with conventional prophylaxis showed comparable rates of GVHD, relapse and overall survival (48). Whether these results will hold up in the setting of an RCT has been tested in the recently completed multi-center RCT (NCT02345850) comparing *ex-vivo* CD34 selection to PTCy + MMF and conventional Tac/MTX prophylaxis, the results of which are eagerly awaited.

The other notable issue with pan-TCD has been a higher incidence of graft failure and slower immune reconstitution (IR) leading to higher rates of infectious complications, particularly viral infections (CMV, EBV) (49). The use of additional *in-vivo* TCD in the form of anti-thymocyte globulin (ATG), particularly in the haploidentical setting can potentially be helpful in achieving better engraftment rates (50). Other strategies to improve IR have incorporated direct T-cell add back strategies post stem-cell infusion (51) or the use of megadoses of CD34 selected cells (Perugia group) which seem to have a tolerizing effect (52).

Given these issues with pan-TCD, more selective methods of T-cell depletion aimed at preventing GVHD while preserving GVL are being explored with the availability of sophisticated clinical grade cell separation techniques.

##### Selective T-Cell Depletion Strategies

While a number of strategies have been attempted for selective TCD, they did not meet with lasting success. Depletion of CD5+ T-cells and CD8+ T-cells were tried in the 1990s; while rates of GVHD were encouraging, rates of relapse were high leading to the abandonment of these strategies. CD6 depletion is separately discussed in the section on T-cell modulation.

More recently Bleakley et al. have looked at CD45RA (naïve) TCD with the understanding that it is primarily the naïve T-cells in an allograft that are alloreactive. Unfortunately, this strategy has not produced encouraging results thus far. Bleakley et al. reported a first in human trial where they performed CD45RA (naïve) TCD via a two-step immunomagnetic bead-based procedure in 35 adult patients. Although 34/35 patients engrafted, rates of aGVHD were high in the 66% range. This was not improved when naïve TCD was combined with a/b TCD (described below) either and this approach is not widely used at this time.

##### α/β TCD

While a majority of T-cells express α/β receptors T-cell receptors (TCR), 2–10% of T-cells express γ/δ TCR. These γ/δ T-cells are believed to have important innate immune effects characterized by rapid cytokine release and killing of viral infected and tumor cells (53). This makes them an attractive candidate to potentially mediate GVL without inducing GVHD by the selective depletion of α/β T-cells. In a prospective single-arm pediatric trial in patients with acute leukemia, an encouraging GRFS of 70% was seen (54). The median follow-up for surviving patients in this study was 46 months. This approach is being tested in a number of other trials in pediatric and adult patients; in one of these a CNI-free GVHD prophylaxis strategy for acute leukemia patients undergoing 1–2 locus MMUD MAC HSCT (NCT03717480) is being looked at.

#### Modulating T-Cell Co-stimulatory/Co-inhibitory Pathways

During T-cell activation, following initial engagement of antigen by the TCR, a number of co-stimulatory and co-inhibitory signals come into play mediated by receptors on T-cells and APCs. This is true in acute GVHD as well. Hence the modulation of these co-stimulatory and co-inhibitory interactions is one of the new frontiers in the prophylaxis of GVHD.

##### CD28/CTLA-4 Blockade: Abatacept

The most promising of these has been blockade of the CD28/CTLA-4 axis. CD28 and CTLA-4 are both receptors on the T-cell which bind to B7-1/CD80 and B7-2/CD86 ligands on the APC; however, while CD28 is co-stimulatory, CTLA-4 is co-inhibitory. Abatacept (CTLA4-Ig) is the soluble extra-cellular portion of CTLA-4 complexed with immunoglobulin heavy chain which blocks CD28 and CTLA-4, with more of an effect on CD28 leading ultimately to an inhibitory signal. Murine models from Blazar et al. showed that CD28/CTLA-4 blockade could reduce aGVHD lethality (55). Kean et al. performed a promising feasibility study in humans and reported results from a phase II RCT comparing standard of care (SOC) +abatacept to abatacept only in pediatric and adult patients. Grades III-IV aGVHD were significantly decreased and OS was improved in the abatacept arm (56). These impressive results have led to FDA breakthrough designation for this drug.

Since this approach blocks both stimulatory and inhibitory pathways, the concern for unwanted T-cell activation has been raised; hence more selective approaches to blocking CD28 are also being investigated. As an example, FR104 (CD28-specific pegylated-Fab') with and without sirolimus are being investigated in non-human primate models (57).

#### Enhancing Regulatory T-Cells

Tregs (CD4+CD25+Foxp3+) comprise a unique subset of T-lymphocytes that can be derived from the thymus or converted from CD4+CD25- cells (inducible or iTregs). Tregs play an important role in immune homeostasis and a favorable balance between Tregs and effector T-cells may be important to prevent GVHD. While *ex-vivo* expansion of Tregs is possible (58), there are concerns about their stability *ex-vivo*. Some preliminary data in alternative donor transplantation has shown that infusion of such expanded Tregs can be beneficial (59).

Hence, rather than the direct infusion of Tregs, other approaches have been attempted which can upregulate Tregs or enhance their functionality in the post-transplant immune milieu. One such approach involves invariant natural killer T (iNKT) cells.

##### Invariant Natural Killer T Cells

iNKT cells are unique in that they co-express both T and NK cell markers and therefore straddle both the innate and adaptive immune system with a semi-invariant TCR that recognizes glycolipid antigens presented by the major histocompatibility complex (MHC) class I-like molecule CD1d. They modulate the immune system via IL-4 and IL-10. In murine models, iNKT cells reduced GVHD both by a switch to a Th2 cytokine profile and/or IL-4-dependent Treg expansion. These mice were conditioned with a regimen incorporating total lymphoid irradiation plus ATG (TLI-ATG) (60, 61). This was then translated in a proof of concept study in humans with promising GVHD outcomes (62). An analysis of post-transplant immune reconstitution showed that low iNKT/T cell ratios were independently associated with rates of acute GVHD (63) while another provocative study suggested that the larger numbers of iNKT cells in the donor graft correlated with improved GVHD free relapse free survival (64). Direct infusion of *ex-vivo* expanded iNKT cells is also an area of investigation.

In this contect, REGiMMUNE is a compound in which KRN7000, a synthetic alpha-galactosylceramide derivative and a CD1d ligand, is embedded in a lipid bilayer. REGiMMUNE has been shown to reduce aGVHD mortality by expanding Tregs via iNKT cells in murine models (65). In a Phase IIa trial REGiMMUNE in combination with sirolimus did reduce overall and acute GVHD although more mature data is awaited (66).

#### Targeting T-Cell Trafficking

##### Vedolizumab

Alloreactive CD8+ T-cells bound for the intestines express high levels of integrin β7 (α4β7) that binds to its ligand mucosal addressin cell adhesion molecule 1 (MAdCAM 1) in Peyer's patches and gut-associated lymphoid tissue (GALT) in the intestinal mucosa. Vedolizumab is a humanized moAb which prevents T-cell trafficking to the gut by targeting α4β7integrins on the T-cells. Early proof-of-concept and restrospective analyses have shown promising efficacy with vedolizumab in steroid refractory aGVHD (67). Given its effect on T-cell trafficking, an early critical event in GVHD pathogenesis, it was then tested in the context of prophylaxis in a phase 1b study where it was moderately safe with low rates of acute and chronic GVHD (68). A phase III RCT comparing vedolizumab + SOC prophylaxis to SOC is currently underway (NCT03657160).

##### Maraviroc

Maraviroc is an antagonist of CCR5, a chemokine receptor that has been implicated in T-cell trafficking during GVHD pathogenesis. Maraviroc appears to block lymphocyte chemotaxis without actually affecting T-cell function which made it an attractive candidate as a prophylactic agent. However, in a prospective non-randomized study from the BMT CTN, maraviroc in combination with standard Tac/MTX was not superior to standard of care and in this trial the PTCy/Tac/MMF arm fared the best (21).

#### Targeting Cytokine Pathways

##### Tocilizumab

Interleukin-6 (IL-6), an inflammatory cytokine has been shown to be one of the chief mediators of aGVHD in murine models (69). Therefore, IL-6 blocking agents could prevent aGVHD. In a phase II trial, tocilizumab, a humanized monoclonal antibody against the IL-6 receptor (IL-6R) was found to be promising (70); however, in a placebo-controlled phase III study from Australia, there was no significant difference in grades II-IV or III-IV aGVHD (71). This is a salient reminder that given the complex pathophysiology of aGVHD, with crosstalk between myriad cytokines and immune effector cells, targeting multiple cytokine pathways will be required for efficacy. These translational advances are summarized in Table 1.

**Table 1:** Translational advances and experimental strategies in GVHD prophylaxis.

	**Translational advances in GVHD Prophylaxis**		**Experimental GVHD prophylaxis strategies**	
1.	*In vivo* T-cell depletion/modulation		Targeting tissue damage/endothelial damage	
A.	Post-transplant cyclophosphamide	Phase II/III	Siglecs/CD24 Fc	Murine models
B.	Anti-thymocyte Globulin	Phase III	Defibrotide	Ongoing Phase II
C.	Sirolimus	Phase III		
2.	*Ex-vivo* T-cell depletion/modulation		T-cell modulation	
A.	Pan T-cell depletion	Phase II/III	Notch pathway	Pre-clinical
B.	Selective T-cell depletion		Mesenchymal stem-cells	Exploratory
	a/b T-cell depletion	Phase I/II		
	CD45RA (naïve) T-cell depletion	Phase I/II		
3.	Modulating T-cell co-stimulatory/inhibitory pathways		Modulating T-cell co-stimulatory/inhibitory pathways	
A.	CD28/CTLA-4 blockade/Abatacept	Phase III	OX40L blockade	Murine/non-human primate models
			ALPN101	Murine models, Phase I/II ongoing
			CD6 blockade: Itolizumab	Xenograft models, Phase I/II ongoing
4.	Enhancing Tregs (regulatory T-cells)		Targeting T-cell trafficking	
	iNKT-cells	Murine models, proof of concept in humans	PSGL-1	Murine models
	REGimmune	Phase IIa		
5.	Targeting T-cell trafficking		Subset T-cell depletion	
	Vedolizumab	Phase 1b, Phase III ongoing	Xenikos/T-guard	Exploratory
	Maraviroc	Phase III (negative study)		
6.	Targeting cytokine pathways		Targeting cytokine pathways	
	Tocilizumab	Phase III (negative study)	AAT (Alpha-1 antitrypsin)	Phase II/III ongoing
			JAK-STAT	Phase I ongoing

### Novel GVHD Prophylactic Strategies on the Horizon

There are novel therapies which have not yet been successfully translated to clinical practice but hold great promise. These therapies are based on innovative targets based on a more intricate understanding of the pathophysiology of GVHD.

#### Targeting Tissue Damage/Endothelial Injury

##### Siglecs/CD24 Fc

As mentioned above in the section on the immunobiology of GVHD, conditioning regimen associated tissue damage exposes antigens which comprise pathogens or Pathogen-Associated Molecular Patterns (PAMPs) and components of damaged cells (Danger-Associated Molecular Patterns or DAMPs) which trigger activation of the innate immune system. Conversely Sialic-acid-binding-immunoglobulin-like lectins (Siglecs) are a particular class of pattern recognition receptors that downregulate innate immune responses (72). A number of Siglec homologs have been identified in mice and humans and are all characterized by immunoreceptor tyrosine-based inhibitory motifs (ITIMs) or ITIM-like regions in their intracellular domains.

A role for Siglecs in modulating adaptive T-cell mediated immune responses has also been proposed. Reddy et al. have shown that Siglec-G interacts with CD24c in murine models and this interaction CD24 suppresses TNF-α, IL-1β, and IL-6 via NFkB and therefore is promising in the domain of GVHD prophylaxis (73).

##### Defibrotide

Defibrotide is a polydisperse mixture of predominantly single-stranded polydeoxyribonucleotides which in pre-clinical and human studies has demonstrated profibrinolytic, antithrombotic, anti-inflammatory and angio-protective effects ultimately resulting in stabilization of endothelial cells (74). Defibrotide is used in the treatment of veno-occlusive disease/sinusoidal obstruction syndrome (VOD/SOS), another morbid complication of allogeneic transplantation. Endothelial activation is also associated with transplant conditioning regimens and prime the host for GVHD. In a randomized phase II pediatric trial of defibrotide in VOD/SOS, the incidence and severity of aGVHD at days +30 and +100 were significantly better in the defibrotide-treated arm in patients who underwent HSCT (75). This signal is being further explored in a phase II randomized, open-label study comparing defibrotide + SOC vs. SOC alone in pediatric and adult patients for the prevention of aGVHD (NCT03339297).

#### T-Cell Modulation

##### Notch Pathway

The canonical NOTCH pathway has been shown to play a critical role in T-cell activation, differentiation, and function in aGVHD pathogenesis (5). Using humanized monoclonal antibodies, it has now been shown that Notch-deprived T-cells produce less inflammatory cytokines but proliferate normally, with a preferential increase in Tregs, without compromising GVL, mediated chiefly by NOTCH1, and Dll-4 (6). Selective NOTCH blockade is an exciting new frontier and offers potential for clinical translation.

##### Mesenchymal Stem-Cells

Attempts have been made to utilize the immunomodulatory properties of MSCs for GVHD prophylaxis based on murine models of HLA-mismatched transplantation with co-transplantation of hematopoietic stem-cells and MSCs (76). Ning et al. showed in a small randomized trial, the incidence of grade II-IV aGVHD was 11.1% in the MSC group compared to 53.3% in the control group (77). However, the sample size in this trial was very small (*n* = 25) and larger studies are needed to further study the effect of MSCs on preventing GVHD. A randomized phase II trial has also shown some beneficial effect on cGVHD and need to be further studied (78).

#### Targeting T-Cell Co-stimulatory Pathways (CD24 Fc)

##### OX40L Blockade

OX40 (CD134) is a co-stimulatory receptor found on T-cells while its ligand OX40L is expressed on dendritic cells, B-cells, and endothelial cells. In 2003 Blazar et al. investigated the OX40 regulation of GVHD in murine models and found that antagonistic anti OX40L moAb or the use of OX40^−/−^donor or recipient mice resulted in similar reduction in GVHD (79). Further, although OX40 was expressed on CD4 and CD8 cells, the effect of OX40 appeared to be mediated chiefly by CD4+ cells. OX40 is also a strong negative regulator of Foxp3(+) Tregs (80) and therefore blockage could enhance Treg reconstitution which could be beneficial in GVHD. Tkachev et al. (39) then have shown that in non-human primate models, the combination of KY1005 (OX40L blocking antibody) and sirolimus has synergistic activity in reduction of GVHD mortality associated with control of both Th/Tc1 and Th/Tc17 activity. In addition, there was a Treg sparing effect with the combination. This exciting approach is now being translated to the clinic.

##### ALPN101

Inducible Costimulator (ICOS) is a member of the CD28/CTLA-4 family expressed on activated T-cells while the ICOS ligand (ICOSL), a B7 family member is constitutively expressed on B-cells, macrophages and dendritic cells and upregulated on APCs *via* TNF-alpha and lipopolysaccharides (LPS). In murine models of GVHD, blockade of the ICOS:ICOSL interactions via moAb or ICOS^−/−^ mice resulted in significant decrease in GVHD and GVHD related mortality both mediated by CD4+ and CD8+ cells (81).

ALPN101 is a novel Fc fusion protein of a human ICOSL variant immunoglobulin domain (single domain vIgD™) binding both ICOS and CD28 at higher affinity than wild-type molecules, designed to inhibit both the CD28 and ICOS pathways to dampen co-stimulatory responses during alloreactive T-cell activation. In a murine dose-ranging study, ALPN101 inhibited GVHD responses at all doses and more significantly than the comparator belatacept, an approved CTLA-4-Fc protein CD28 pathway inhibitor (82).

A phase 1/2 dose finding study (BALANCE) is ongoing in patients with steroid sensitive and steroid refractory aGVHD (NCT04227938) investigating this potentially transformative effect and this is another promising target for GVHD prophylaxis.

##### CD6 Blockade: Itolizumab

CD6 is a co-stimulatory receptor on T-cells that binds to activated leukocyte cell adhesion molecule (ALCAM), a ligand on APCs and is involved in T-cell activation and trafficking. Historically CD6 T-cell depletion using a monoclonal antibody (moAb) was evaluated in a single-arm trial (*n* = 112) with aGVHD rates in the 18% range using bone marrow grafts (41). More recently, itolizumab, a humanized anti-CD6 moAb was tested in xenograft models with some evidence that it could modulate T-cell activity (83). This molecule has also been fast-tracked by the FDA and is being tested in a phase I/II study for first-line treatment (with steroids) of severe aGVHD (NCT03763318) and may have a role in prophylaxis.

#### Targeting T-Cell Trafficking

##### PSGL-1

P-selectin is one of a family of three glycosylated lectins (E, L, and P-selectin) which is constitutively expressed on the vascular endothelium of skin and bone marrow and inducibly expressed on other cells during inflammation. P-selectin is a receptor for PSGL-1, a glycoprotein strongly expressed on all leukocytes (84). PSGL-1 mRNA has been shown to be upregulated during GVHD in experimental models (85). P-selectin deficient mice were shown to have less GVHD morbidity and mortality; in addition T-cells were redirected from Peyer's patches and GALT to spleen and lymph nodes indicating that disruption of P-selectin interactions during GVHD pathogenesis can affect T-cell trafficking to target organs (84). Although it is likely that disruption of this pathway alone may not fully abrogate selectin interactions in GVHD, it is a promising new target.

#### Targeting Cytokine Pathways

##### Alpha-1 Antitrypsin

AAT is a liver derived serine protease inhibitor which can inhibit proinflammatory plasma cytokines and induce anti-inflammatory IL10 among other somewhat protean immunologic functions. It has also been shown to be involved in the *in-vivo* induction of Treg. In preclinical aGVHD models, AAT reduced inflammatory cytokines, altered the ratio of effector and regulatory T-cells and reduced levels of DAMPs (86). AAT has shown promise in early phase trials for SR aGVHD (87) and is being tested in phase III trials. This drug is also being tested in the prophylactic setting in a phase II/III randomized, multi-center, placebo-controlled study for prevention of aGVHD (NCT03805789).

##### JAK-STAT

The Janus kinase family comprises intra-cellular signaling proteins (JAK-1, 2, 3, and tyrosine kinase 2) involved in downstream transduction of various cytokine pathways (88). They are fundamentally involved in all three phases of GVHD pathogenesis by regulating the activity of APCs, T- and B-lymphocytes (89). Pre-clinical studies showed that JAK-1/JAK-2 could reduce GVHD without affecting GVL (90, 91) including an effect on T-cell trafficking and enhancement of Tregs. JAK-1/JAK-3 inhibition also appears to reduce GVHD in murine models (92). Following on encouraging early phase studies with JAK-1/JAK-2 inhibitor ruxolitinib (Rux) in SR aGVHD, phase III data has now been reported (Rux vs. investigator's choice for SR aGVHD, REACH-2) which shows better overall response rates with Rux although a benefit in NRM could not be shown. JAK inhibition could be an exciting new frontier for GVHD prophylaxis as well. Choi et al. (93) showed that baricitinib (JAK1/JAK2 inhibitor) completely prevented GVHD in murine models without hampering GVL by multiple mechanisms including expansion of Tregs by preserving JAK3-STAT5 signaling; downregulation of CXCR3 and helper T cells 1 and 2.

So far clinical data is limited to a pilot study in myelofibrosis patients where a combination of ATG, ruxolitinib, and PTCy was used as GVHD prophylaxis with acceptable engraftment rates (94) and a single-arm study where Rux was used to replace CNIs in patients with CNI intolerance (95). Itacitinib, a selective JAK-1 inhibitor is being investigated in combination with CNI for primary prophylaxis of GVHD (GRAVITAS-119 trial, NCT03320642).

#### Subset TCD

##### Xenikos/T-Guard

Monoclonal antibodies conjugated with immunotoxins is a method of selective TCD that has been attempted in the past with CD5 as a target among others as described above. T-guard is a immunotoxin combination comprised of a mixture of anti-CD3 and anti-CD7 antibodies separately conjugated to recombinant ricin A (CD3/CD7-IT), which induces *in vivo* depletion of T cells and natural killer (NK) cells and suppresses T cell receptor activation.

This was first evaluated in a phase I/II trial in humans in SR aGVHD with a 50% response rate and manageable toxicities albeit with evidence of capillary leak and thrombotic microangiopathy (96). This may be a potential target for prophylaxis in the future. These experimental methods are summarized in Table 1.

### Prevention of cGVHD

Although cGVHD is a distinct clinical and immunologic entity from aGVHD, there are limited interventions that specifically target the prevention of cGVHD. In general we know that patients who have less aGVHD will likely get less cGVHD and so the prevention of aGVHD is important in the prevention of cGVHD. In terms of donor and transplant related interventions, younger same-sex donors and the use of bone marrow product rather than PBSC has been shown to reduce rates of cGVHD (97).

T-cell directed approaches that have been quite successful include ATG and more recently PTCy as outlined above. B-cell directed approaches are an area of interest given our current understanding of the important role that B-cells play in the pathophysiology of cGVHD. Rituximab, a monoclonal antibody targeting CD20 was evaluated in a phase II trial with cGVHD rates in the 48% range cGVHD requiring immunosuppression in the 31% range (98). This was promising at the time and led to an ongoing randomized trial where obinutuzumab, another monoclonal B-cell directed antibody is being tested for cGVHD prevention (NCT02867384). It will be interesting to see how it will fare in comparison to PTCy.

Another area of interest is the augmentation of tolerance by the use of low-dose IL-2 (aldeskeukin) to enhance Treg reconstitution creating a favorable immunologic milieu for the prevention of cGVHD. This strategy has had success in the therapy of steroid-refractory cGVHD (99) and may have a role in prevention as well although remains investigational at this time.

## Discussion

GVHD prophylaxis has evolved over the last few decades from direct *in-vivo* and *ex-vivo* pan T-cell depletion strategies to more directed immunomodulatory strategies based on a more comprehensive understanding of GVHD immunobiology. Nevertheless, the basic backbone of CNI based prophylaxis has survived the test of time. We have touched upon this current standard in this review and further discussed important translational advances and exciting pre-clinical strategies which may be a part of future prophylactic regimens.

Of these translational advances, PTCy is arguably the most exciting and a potential replacement for standard CNI/MTX based prophylaxis in multiple transplant settings. Sometimes considered to be an elegant method of *in vivo* T-cell depletion, mechanistic studies have indicated that PTCy has a far more complex impact on the post-transplant immune system including a Treg sparing role which is of great interest in the community (20). In haploidentical transplants, PTCy based GVHD prophylaxis is the standard, typically incorporating a CNI and MMF in the most widely used regimens. Although rates of grades II-IV aGVHD are comparable in haploidentical transplants with PTCy based prophylaxis compared to CNI/MTX prophylaxis in MUD transplants, the rates of severe acute and chronic GVHD are far lower without significantly compromising relapse rates which makes it a very attractive strategy (100). In fact, the results in the haploidentical setting with PTCy based prophylaxis have been so impressive that this platform is now being tested in the HLA_matched setting where it is being compared to standard CNI/MTX based prophylaxis. Data from a small RCT (22) as well as a larger prospective trial (BMT CTN 1203) (21) in the reduced intensity setting have already generated encouraging signals where the PTCy arm performed better than the standard CNI arm as well as other potential novel strategies. Based on this data, some centers have already migrated to using PTCy based prophylaxis for matched unrelated donor and in some cases even in matched related donor transplantation. However, from a purist's viewpoint, data from a well-powered RCT is still not sufficient to change practice standards and the results of BMT CTN 1703 comparing PTCy based prophylaxis to CNI/MTX are eagerly awaited. In the myeloablative setting, the standard remains CNI/MTX although PTCy based prophylaxis will likely be tested in this setting as well. Other *in-vivo* TCD strategies such as ATG are still widely used, although the most profound effect of ATG appears to be on severe chronic GVHD and comes at the cost of poorer T-cell immune reconstitution and therefore more infectious complications. Sirolimus is another drug that has had promising results in reducing severe acute GVHD without much impact on chronic GVHD and is a reasonable alternative to CNI/MTX (34).

In the domain of *ex-vivo* TCD, pan TCD is still performed routinely in certain centers; once again with gains in the realm of severe chronic GVHD at the cost of more infectious complications. There have been some concerns about higher rates of relapse with *ex-vivo* TCD as well. These three important methods of T-cell depletion for GVHD prophylaxis, namely CNI/MTX, PTCy and *ex-vivo* pan-TCD have been compared in a multi-center RCT (BMT CTN 1301), the results of which are eagerly awaited as well. In the last decade the spotlight has shifted to methods of selective *ex-vivo* TCD with limited success in the clinical setting. a/b TCD which attempts to reduce GVHD without affecting GVL and can be performed without the use of post-transplant immunosuppression is promising and may be an important modality in the future.

Separate from direct TCD (*in-vivo* or *ex-vivo*), a new frontier in GVHD prophylaxis is targeting immune checkpoints which regulate T-cell activation. Given the dramatic success of checkpoint inhibitors in the world of solid tumor oncology, there has been tremendous interest and a much better understanding of these checkpoints in recent years. In the case of GVHD of course, researchers have tried to downregulate rather than upregulate T-cell activation following initial antigen engagement by the T-cell receptor complex. Although there are a number of molecules being tested at the bench and detailed in this review, the most promising of these has been blockade of the CD28/CTLA-4 axis with Abatacept (CTLA-Ig) with an eventual inhibitory signal downstream to the T-cell. Results from a RCT with pediatric and adult patients has shown a dramatic reduction in severe acute and chronic GVHD including in mismatched unrelated donors (56). With FDA breakthrough status, this molecule has the potential to be an integral part of GVHD prophylaxis in the future although it is unclear if it is more effective than PTCy based prophylaxis. Certainly cyclophosphamide, a drug that has been used for decades, is far more affordable and therefore a platform easily generalizable in more resource poor settings.

Targeting T-cell trafficking, an early event in GVHD prophylaxis, is being tried with integrin blockers such as vedolizumab. It is logical that inhibiting the very movement of effector T-lymphocytes to target organs should better prevent GVHD rather than trying to arrest the widespread inflammation and cytokine cascades which characterize the final common pathway in GVHD pathogenesis. As a testimony to that, when tocilizumab an IL-6 blocker was evaluated, despite promising phase II single-arm data, was not more effective than standard prophylaxis in a phase III RCT (71). This has in fact been the case with numerous promising prophylactic therapies which perform well in single-arm studies but have not been a home run in well-designed phase III studies.

Within the limitations of this review, we have highlighted some of the exciting pre-clinical science that has the potential to translate into effective prophylactic therapies which target GVHD pathogenesis beyond direct T-cell depletion. Targeting the interaction between DAMPs/ PAMPs on APCs and Siglecs with a downstream inhibitory effect on cytokine cascades as well as investigating a role for Siglecs in modulating adaptive T-cell mediated immunity are areas of interest (73). Endothelial damage, another inciting pro-inflammatory event in GVHD pathogenesis is being targeted by drugs like defibrotide which have enjoyed tremendous success in the therapy of VOD. Targeting selectin interactions such as PSGL-1 (84) is another developing area in the field of GVHD therapeutics and prophylaxis.

Pathways critical in T-cell modulation during activation and proliferation such as the Notch pathway has been an area of great interest although not ready for translation at this time (6). Bolstered by the success of Abatacept, other molecules targeting checkpoints such as OX40L blockade (including combination with sirolimus) (39), blockade of the ICOS: ICOSL interaction with ALPN101 (81) and CD6 blockade (Itolizumab) (83) are extremely exciting. The role of AAT in prophylaxis both as an immunomodulator as well as in opposing inflammatory cytokines is being looked at.

In conclusion, while GVHD prophylaxis in 2020 still incorporates the traditional paradigms of CNI based prophylaxis, PTCy is knocking on the door and a number of exciting new translational therapies and pre-clinical advances are on the horizon which promise to challenge the established paradigms.

## Author Contributions

JHA supervised and edited manuscript along with MG. All authors contributed to the article and approved the submitted version.

## Conflict of Interest

The authors declare that the research was conducted in the absence of any commercial or financial relationships that could be construed as a potential conflict of interest.
